# Evolutionary implications of *NOTCH2NLC* mutations: brain structural changes in neuronal intranuclear inclusion disease revealed by comprehensive morphometry

**DOI:** 10.1093/braincomms/fcag206

**Published:** 2026-06-03

**Authors:** Si Shen, Hong-Fei Tai, Songtao Niu, Hua Pan, Xingao Wang, Bin Chen, Yuzhi Shi, Hengheng Wang, Shan Lv, Yaou Liu, Zaiqiang Zhang

**Affiliations:** Department of Neurology, Beijing Tiantan Hospital, Capital Medical University, Beijing 100070, China; Department of Neurology, Beijing Tiantan Hospital, Capital Medical University, Beijing 100070, China; National Clinical Research Center for Neurological Diseases, Beijing 100070, China; Department of Neurology, Beijing Tiantan Hospital, Capital Medical University, Beijing 100070, China; National Clinical Research Center for Neurological Diseases, Beijing 100070, China; Department of Neurology, Beijing Tiantan Hospital, Capital Medical University, Beijing 100070, China; National Clinical Research Center for Neurological Diseases, Beijing 100070, China; Department of Neurology, Beijing Tiantan Hospital, Capital Medical University, Beijing 100070, China; National Clinical Research Center for Neurological Diseases, Beijing 100070, China; Department of Neurology, Beijing Tiantan Hospital, Capital Medical University, Beijing 100070, China; National Clinical Research Center for Neurological Diseases, Beijing 100070, China; Department of Neurology, Beijing Tiantan Hospital, Capital Medical University, Beijing 100070, China; National Clinical Research Center for Neurological Diseases, Beijing 100070, China; Department of Neurology, Beijing Tiantan Hospital, Capital Medical University, Beijing 100070, China; National Clinical Research Center for Neurological Diseases, Beijing 100070, China; Department of Radiology, Beijing Tiantan Hospital, Capital Medical University, Beijing 100070, China; Tiantan Image Research Center, National Clinical Research Center for Neurological Diseases, Beijing 100070, China; Department of Radiology, Beijing Tiantan Hospital, Capital Medical University, Beijing 100070, China; Tiantan Image Research Center, National Clinical Research Center for Neurological Diseases, Beijing 100070, China; Department of Neurology, Beijing Tiantan Hospital, Capital Medical University, Beijing 100070, China; National Clinical Research Center for Neurological Diseases, Beijing 100070, China

**Keywords:** neuronal intranuclear inclusion disease, human-specific gene, evolutionary implications, voxel-based morphometry, surface-based morphometry

## Abstract

This study investigated brain structural changes associated with *NOTCH2NLC* gene mutations in neuronal intranuclear inclusion disease (NIID) patients, focusing on the evolutionary implications of this human-specific gene in brain development. We analysed 41 NIID patients and 21 healthy controls using voxel-based morphometry and surface-based morphometry to assess differences in grey matter volume and cortical complexity. Spatial relationships between brain atrophy and white matter hyperintensity volume as well as cerebrospinal fluid fraction were examined. Additionally, we conducted exploratory Spearman correlation analyses to evaluate associations between regional grey matter volume and clinical variables, including GGC repeat length, disease duration, age at onset and cognitive scores. NIID patients exhibited extensive reductions in grey matter volume and cortical thinning in multiple brain regions, with pronounced effects in the prefrontal cortex and cerebellum. The parietal lobe, insula and posterior cingulate gyrus showed decreased gyrification index and fractal dimension, while certain regions of the temporal and frontal lobes showed increased gyrification index and fractal dimension. Furthermore, in the NIID group, white matter hyperintensity volume and cerebrospinal fluid fraction were negatively correlated with grey matter volume in the olfactory cortex, orbital gyrus, anterior cingulate gyrus, insula, amygdala and temporal pole. Exploratory analyses suggested that longer GGC repeats were associated with greater atrophy in the striatum, middle cingulate cortex, sensorimotor cortex and cerebellum; earlier age at onset with thalamic (mediodorsal/pulvinar), occipital and cerebellar atrophy; and poorer cognitive scores with atrophy in the anterior cingulate cortex, superior occipital gyrus and superior temporal pole. This study uncovers widespread and complex cerebral structural changes in NIID patients, predominantly affecting the prefrontal cortex, cerebellum, insula and limbic system structures. These findings provide new insights into the neuroanatomical basis of NIID and support the hypothesis that human-specific genetic innovations driving cortical expansion may concurrently confer selective vulnerability to neurodegeneration.

## Teaser

Shen *et al*. report that patients with neuronal intranuclear inclusion disease caused by abnormal *NOTCH2NLC* repeat expansion exhibit widespread brain atrophy and altered cortical folding, particularly in prefrontal, cerebellar, insular and limbic regions, supporting the idea that human-specific genes that drove brain expansion may also increase vulnerability to neurodegeneration.

## Graphical Abstract

**Graphical Abstract:**
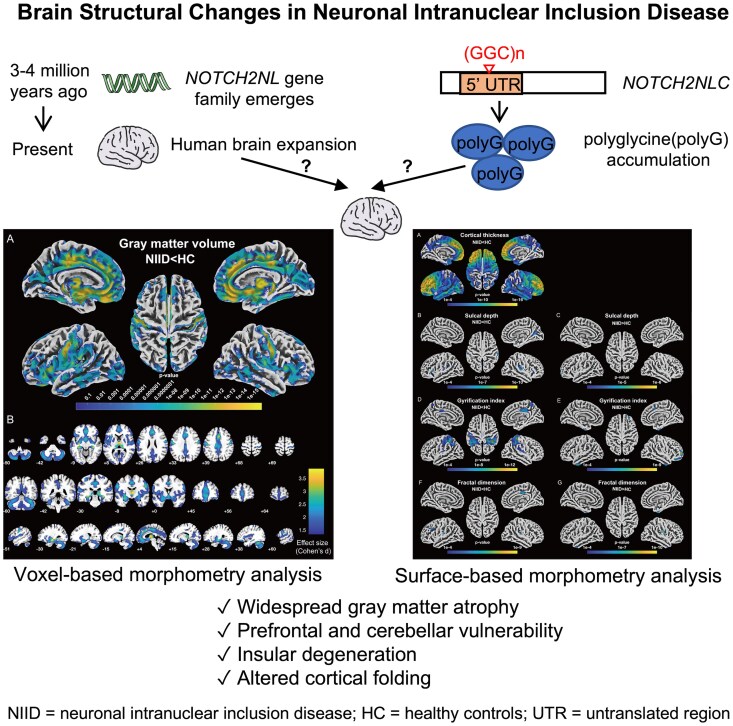

## Introduction

The human brain, particularly the prefrontal cortex, has undergone remarkable expansion and specialization during evolution, distinguishing *Homo sapiens* from other primates.^1,2^ This evolutionary leap is partly attributed to human-specific genes such as *ARHGAP11B*, *SRGAP2* and notably, the *NOTCH2NL* gene family.^3-9^ The *NOTCH2NL* gene family emerged approximately 3–4 million years ago through partial duplication of *NOTCH2*, coinciding with brain expansion in the hominin lineage.^10,11^ The *NOTCH2NL* genes, comprising *NOTCH2NLA*, *NOTCH2NLB* and *NOTCH2NLC*, enhance Notch signalling to regulate neural progenitor proliferation and differentiation, thereby promoting neocortical expansion, particularly in the prefrontal cortex.^10,11^ Specifically, NOTCH2NLA primarily influences the proliferation of basal progenitors in the subventricular and intermediate zones, thereby contributing to upper-layer neuron production. NOTCH2NLB, characterized by a unique signal peptide and EGF domains that directly interact with the Notch ligand DLL1, potently enhances Notch signalling to maintain apical progenitors in the ventricular zone in a proliferative state, thus expanding the progenitor pool and increasing neuronal output. The neurodevelopmental significance of these genes is underscored by their dosage sensitivity. Copy number variations (CNVs) in the 1q21.1 region harbouring *NOTCH2NLA/B* are associated with brain size abnormalities. Deletions result in microcephaly due to reduced progenitor proliferation and premature differentiation, while duplications lead to macrocephaly via enhanced progenitor proliferation and delayed differentiation.^6,10^ In 2019, GGC repeat expansions in the 5′ untranslated region of the *NOTCH2NLC* gene were identified as the genetic cause of neuronal intranuclear inclusion disease (NIID).^12-15^ This finding not only elucidates the genetic basis of NIID but also highlights a potential evolutionary vulnerability, suggesting that the unique role of this human-specific gene may be key to understanding the neuroanatomical features of the disease.

NIID is a progressive neurodegenerative disorder characterized by eosinophilic hyaline intranuclear inclusions in both the central and peripheral nervous systems, as well as in various visceral organs.^16,17^ The clinical spectrum of NIID is remarkably diverse, encompassing cognitive impairment, parkinsonism, tremor, cerebellar ataxia, episodic symptoms (such as stroke-like episodes, encephalitis, unconsciousness, seizures and headaches), peripheral neuropathy, myopathy and autonomic dysfunction.^18-20^ Radiologically, NIID often presents with extensive white matter lesions and ventricular enlargement.^16,21,22^ However, the precise impact of *NOTCH2NLC* mutations on brain structure remains elusive, particularly in relation to the evolutionary adaptations that have shaped the human brain.

To bridge this knowledge gap, we utilized neuroimaging techniques, including voxel-based morphometry (VBM) and surface-based morphometry (SBM), to systematically investigate the structural brain changes in NIID. VBM provides unbiased, voxel-wise analyses of grey and white matter volume differences after spatial normalization and tissue segmentation, thereby identifying specific regional atrophy patterns.^23^ In parallel, SBM allows for the assessment of cortical architecture by quantifying cortical thickness (CT, reflecting neuronal density and laminar organization), sulcal depth (SD, indicating sulcal morphology changes related to atrophy or cortical tension), gyrification index (GI, representing the complexity of cortical folding influenced by neurodevelopmental and neurodegenerative factors) and fractal dimension (FD, capturing the geometric intricacy of the cortical surface).^24,25^ These SBM metrics are sensitive to subtle and complex alterations in cortical integrity, folding and topography, which may signify underlying neurodevelopmental or neurodegenerative disruptions overlooked by volumetric analyses alone.

In this study, we aimed to evaluate the brain structural changes in NIID patients using both VBM and SBM. We compared grey matter volume (GMV) and cortical metrics between NIID patients and healthy controls (HCs) and explored the spatial distribution of brain atrophy in relation to key imaging features such as white matter hyperintensity (WMH) volume and cerebrospinal fluid (CSF) fraction in NIID patients. Furthermore, we conducted exploratory correlation analyses to link these regional structural changes with clinical features, including GGC repeat length, disease duration, age at onset (AAO) and cognitive performance. By investigating the effects of *NOTCH2NLC* gene mutations on brain structure, this research seeks to illuminate the broader implications of human-specific genetic influences on neurodegenerative processes.

## Materials and methods

### Participants and clinical assessment

This study included 41 patients with NIID and 21 HCs. NIID patients were recruited from the Department of Neurology at Beijing Tiantan Hospital, Capital Medical University. The diagnosis of NIID was established based on the following criteria: (i) clinical presentation of characteristic neurological symptoms, including cognitive impairment, parkinsonism, tremor, ataxia, limb weakness, headache, episodic neurological events and autonomic dysfunction; (ii) positive skin biopsy showing eosinophilic intranuclear inclusion bodies, with p62 or ubiquitin antibody staining positive; and (iii) genetic confirmation of pathological GGC repeat expansions (>60 repeats) in the 5′ untranslated region of the *NOTCH2NLC* gene. Inclusion criteria for NIID patients included the following: (i) a definite diagnosis of NIID according to the criteria above; (ii) age ≥18 years; and (iii) ability to complete multimodal MRI and clinical assessments. Exclusion criteria were as follows: (i) major structural brain lesions attributable to non-NIID causes (e.g. cerebral infarction, brain tumours, traumatic brain injury); (ii) comorbidity with other neurological disorders (e.g. Alzheimer’s disease, Parkinson’s disease); and (iii) inability to complete MRI examinations or raw MRI data not meeting quality requirements for analysis. Additionally, 21 age- and sex-matched HCs were recruited. Inclusion criteria for HCs were as follows: (i) no history of neurological or psychiatric disorders; (ii) age ≥18 years; and (iii) ability to complete multimodal MRI and clinical assessments. Exclusion criteria were consistent with those for the NIID group. Clinical data, including sex, age, AAO, disease duration and GGC repeat numbers, were collected. Cognitive function was evaluated using the Mini-Mental State Examination (MMSE) and the Montreal Cognitive Assessment (MoCA). The study was approved by the Ethics Committee of Beijing Tiantan Hospital (Approval No. KY-2020-014-02) in accordance with the Declaration of Helsinki. Informed consent was obtained from all individuals involved.

### MRI acquisition

MRI scans, including 3D T1-weighted images and 3D FLAIR, were performed using a 3.0 T MR scanner (Philips Ingenia CX, Best, Netherlands). To minimize motion artefacts, foam pads and soft head restraints were used to immobilize the head. Participants were instructed to keep their eyes closed and remain as still as possible. Scans were monitored in real-time, and sequences with visible motion artefacts were re-acquired whenever feasible. Sagittal 3D T1-weighted images were acquired using a magnetization-prepared rapid gradient echo (MPRAGE) sequence with the following parameters: Repetition Time (TR)/Echo Time (TE) = 6.6 ms/3 ms; Inversion Time (TI) = 880 ms; flip angle = 8°; image resolution = 1 mm × 1 mm × 1 mm; slice number = 196. For the sagittal 3D FLAIR sequence, an inversion recovery fast spin echo technique was employed with the following settings: TR/TE = 4800 ms/228 ms; TI = 1650 ms; flip angle = 90°; image resolution = 1 mm × 1 mm × 1 mm; slice number = 196.

### Data processing

The 3D T1-weighted images were converted from DICOM to NIFTI format using dcm2niix. Subsequent preprocessing and analysis were conducted using the Computational Anatomy Toolbox (CAT12) implemented in Statistical Parametric Mapping (SPM12) running on MATLAB 2023b. We utilized the standard CAT12 preprocessing pipeline for VBM and SBM. For VBM, processing included spatial registration to Montreal Neurological Institute (MNI) space; tissue segmentation into grey matter, white matter and CSF; bias correction; and spatial normalization. For SBM, the ‘Surface and thickness estimation’ option in CAT12 was used to reconstruct central cortical surfaces for both hemispheres based on the projection-based thickness (PBT) approach.^24^ The surface reconstruction included topology correction, spherical inflation and spherical registration. Cortical features including SD, GI and FD were extracted using the ‘Extract additional surface parameters’ module in CAT12. Quality control was performed after preprocessing for each participant. This involved a two-step procedure: (i) review of the automated CAT12 quality report [weighted average (IQR) ≥ B required] and (ii) visual inspection to identify motion artefacts or segmentation errors. Images that did not pass quality control were excluded from further analysis.

Spatial smoothing was applied using an 8 mm Gaussian kernel for GMV in VBM analysis. For SBM analysis, the cortical thickness images were smoothed with a 15 mm Gaussian kernel, and a 20 mm Gaussian kernel was employed for sulcal depth, gyrification index, and fractal dimension.

### ROI-based cortical thickness analysis

To identify the most significantly affected cortical atrophy regions in NIID, we employed CAT12 to extract ROI-based cortical thickness values using the Desikan–Killiany atlas. The Desikan–Killiany atlas was selected for its extensive use and validation in neurodegenerative disease research, providing an optimal balance between regional specificity and statistical power across samples of this size and facilitating comparison with previous studies.^26^ ROC analysis was performed in SPSS to compare cortical thickness between NIID patients and HCs, calculating the area under the curve (AUC) for each brain region to assess discriminatory power. The results were ranked by AUC values and visualized using R. Brain regions were categorized by lobe: frontal, parietal, temporal, occipital, insular and cingulate cortices. Regions where AUC classification did not reach statistical significance were labelled as ‘NS’ and highlighted in grey.

### WMH segmentation

We employed lesion prediction algorithms (LPA) in SPM12 for the segmentation of WMH. T2 FLAIR and T1-weighted images were utilized to achieve the precise identification and segmentation of WMH lesions.

### Volume metrics

Following segmentation of T1-weighted images using the CAT12 toolbox, volumes of grey matter, white matter, CSF and total intracranial volume (TIV) were obtained. Each was divided by TIV to derive the grey matter fraction, white matter fraction and CSF fraction.

### Statistical analysis

Statistical analyses were performed using SPSS software (IBM version 27.0). The Shapiro–Wilk test was used to assess the normality of continuous data distributions. For normally distributed data, we presented results as mean ± standard deviation and compared groups using independent sample *t*-tests. Non-normally distributed data were expressed as median with interquartile range and compared using Mann–Whitney U-tests. Sex distribution was reported as frequency and percentage, with group comparisons performed using Chi-square tests.

For neuroimaging data, we used CAT12 to statistically analyse the smoothed GMV and cortical metrics. First, VBM analysis was conducted using a two-sample *t*-test to evaluate differences in GMV between the NIID and HC groups, adjusting for sex, age and TIV as covariates. Next, SBM analysis was performed using a two-sample *t*-test to compare cortical thickness, sulcal depth, gyrification index and fractal dimension between the two groups, controlling for sex and age. To explore the spatial distribution of GMV associated with WMH volume and CSF fraction in NIID patients, multiple regression analyses were performed, adjusting for sex, age and TIV. We prioritized family-wise error (FWE) rate correction at *P* < 0.05 with a minimum cluster size of 30 voxels. If preliminary analysis revealed no significant results, a more lenient threshold (voxel-level *P* < 0.001, with a minimum cluster size of 30 voxels) was applied for exploratory analysis. For all significant findings, detailed information on anatomical locations, cluster sizes, peak coordinates and statistical values was documented. For VBM results, location information was recorded using the AAL3 atlas, while for SBM results, the Desikan–Killiany atlas was utilized.

To explore the potential clinical relevance of structural brain changes, we performed a ROI-based correlation analysis in the NIID group. We extracted the mean GMV values from AAL3 atlas. Non-parametric Spearman’s rank correlation analyses were conducted to examine the associations between regional GMV and GGC repeat length, disease duration, AAO, MMSE and MoCA scores. Correlation matrices were visualized as heatmaps in GraphPad Prism.

### Power analysis

Given the rarity of NIID and the resulting sample size constraints, a *post hoc* power analysis was conducted using G*Power 3.1 to evaluate the statistical robustness of our findings. Based on the significant clusters identified in the VBM and SBM analysis, the observed effect sizes (Cohen’s *d*) for grey matter volume and cortical metrics differences between NIID patients and HCs ranged from 1.0 to 3.9. With a two-tailed α error probability of 0.05 and the current sample size, the calculated power (1 − β) to detect these structural alterations exceeded 0.80 (range: 0.96–1.00). This indicates that the study possessed sufficient statistical power to identify the characteristic, large-magnitude atrophy patterns associated with NIID.

## Results

### Demographics and structural brain differences

Table 1 presents the demographic and neuroimaging data for all participants. We found no significant differences between the NIID and HC groups in terms of sex and age. However, neuroimaging results revealed significant structural brain differences. The NIID group exhibited significantly greater WMH volumes compared to the HC group (49.919 [28.823–66.581] versus 1.007 [0.586–2.934] mL, *P* < 0.001), highlighting the extensive white matter involvement in NIID. Additionally, the NIID group showed significantly lower grey matter fraction and white matter fraction, alongside a higher CSF fraction (all *P* < 0.001).

**Table 1:** Demographic and imaging features of participants

Variables	NIID (*n* = 41)	HC (*n* = 21)	*P*
Demographics			
Male, *n* (%)	17 (41.46%)	9 (42.86%)	0.916
Age at MRI (years)	63 (59–66)	60 (54–66)	0.267
Age at onset (years)	56 (48–61.5)	−	
Duration (years)	5 (2–10)	−	
GGC repeats	122 (107.5–134.5)	−	
Imaging characteristics			
WMH volume (mL)	49.919 (28.823–66.581)	1.007 (0.586–2.934)	<0.001
GM fraction	0.384 ± 0.030	0.430 ± 0.015	<0.001
WM fraction	0.278 (0.264–0.304)	0.347 (0.327–0.357)	<0.001
CSF fraction	0.339 (0.295–0.359)	0.227 (0.203–0.244)	<0.001

Abbreviations: NIID, neuronal intranuclear inclusion disease; HC, healthy controls; WMH, white matter hyperintensity; GM, grey matter; WM, white matter; CSF, cerebrospinal fluid.

### Grey matter volume analysis

**Figure 1:**
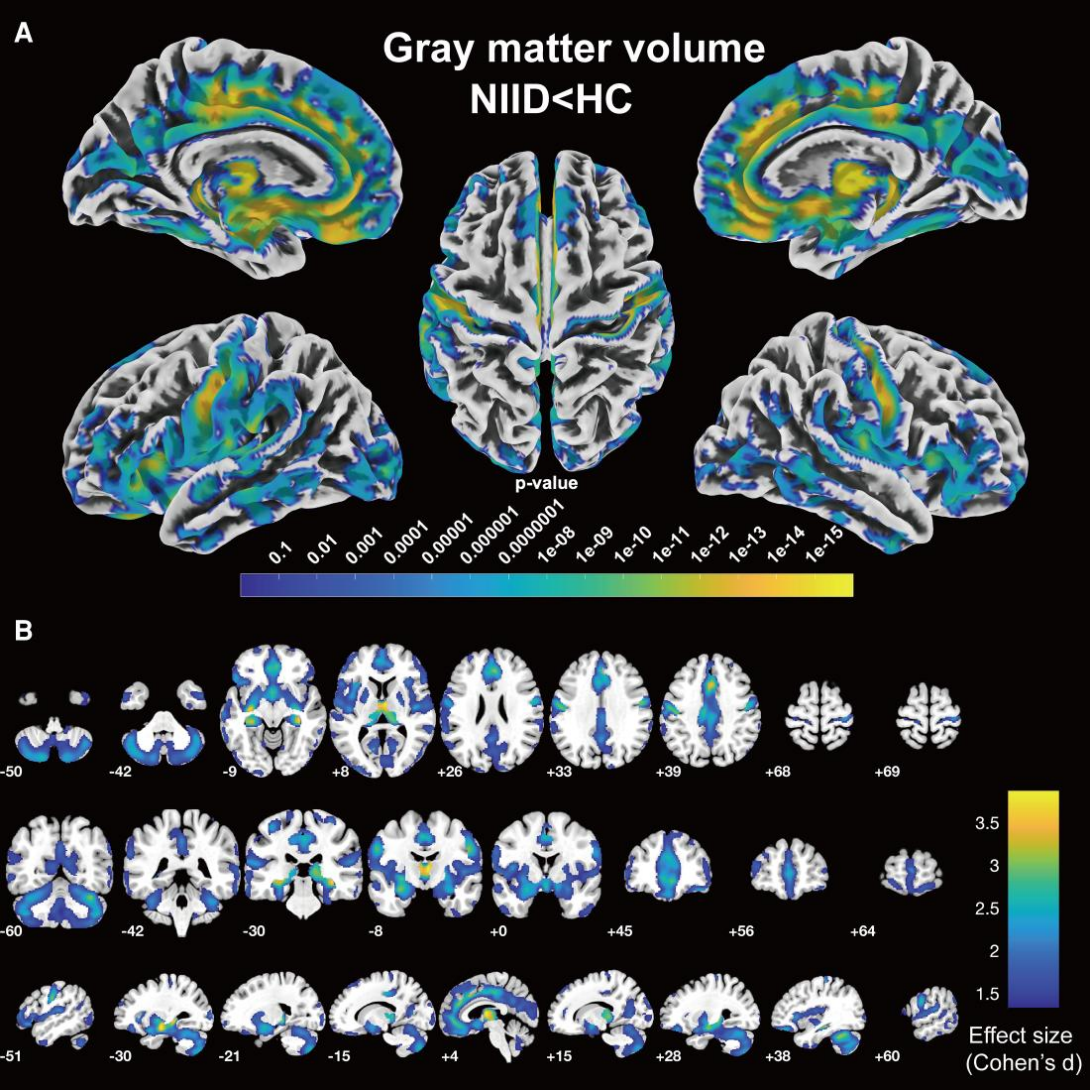
**Grey matter volume reduction in NIID patients.** VBM analysis revealing regions of reduced grey matter volume in patients with NIID compared to HC. (**A**) Surface rendering of significant clusters. (**B**) Representative axial/coronal/sagittal slices in MNI space. Analyses were performed using two-sample *t*-tests with family-wise error (FWE) correction at *P* < 0.05, adjusting for sex, age and total intracranial volume (NIID *N* = 41; HC *N* = 21). NIID, neuronal intranuclear inclusion disease; HC, healthy controls.

Compared to HCs, patients with NIID exhibited significant reductions in GMV in multiple brain regions (FWE corrected, *P* < 0.05), as illustrated in Fig. 1 and detailed in Table 2 and Supplementary Table 1. Notable GMV decreases were observed in the cerebellum, including bilateral Crus I, Crus II, Lobule VI, Lobule VIII and the right Lobule IX and X. Additionally, the bilateral hippocampi, left putamen and portions of the cingulate cortex, particularly the left anterior cingulate and bilateral mid-cingulate and paracingulate, showed significant volume loss. Extensive cortical atrophy was also noted, especially on the medial surfaces, with both insular lobes showing decreased GMV. In the frontal lobe, GMV reductions affected the bilateral superior frontal gyri (medial, orbital and dorsolateral parts), bilateral triangular parts of the inferior frontal gyri, bilateral Rolandic operculum, right middle frontal gyrus, left anterior orbital gyrus and the left supplementary motor area. The parietal lobe displayed reductions in the bilateral postcentral gyri, bilateral precuneus, left paracentral lobule, right superior parietal lobule and right inferior parietal lobule. Temporal lobe changes included the bilateral superior and middle temporal gyri, left inferior temporal gyrus and the temporal pole of the left middle temporal gyrus. In the occipital lobe, GMV reductions were noted in the bilateral calcarine fissures and surrounding cortices.

**Table 2:** Voxel-based morphometry results: brain regions demonstrating significant differences in grey matter volume between NIID patients and healthy controls

Cluster	Peak anatomical region	Cluster size	*T* value	Cohen’s d	*P*-value	Peak MNI coordinates		
						*x*	*y*	*z*
GMV: NIID < HC (FWE corrected, *P* < 0.05)								
1	Unknown (mixed regions)	124 645	14.5	3.9	3.20E−15	4	−8	9
2	Left middle frontal gyrus	103	6.57	1.8	2.70E−07	−30	56	26
3	Left inferior temporal gyrus	484	6.54	1.7	2.90E−07	−51	0	−42
4	Left superior frontal gyrus-dorsolateral	226	6.43	1.7	3.70E−07	−21	64	−9
5	Right angular gyrus	201	6.22	1.7	6.00E−07	60	−60	33
6	Left postcentral gyrus	175	6.19	1.7	6.50E−07	−15	−30	69
7	Right lobule IX of cerebellar hemisphere	99	5.97	1.6	1.10E−06	15	−42	−50
8	Right superior frontal gyrus-dorsolateral	39	5.75	1.5	1.80E−06	28	45	39
GMV: NIID > HC (uncorrected, *P* < 0.001)								
9	Right middle frontal gyrus	118	4.8	1.3	6.20E−06	32	21	44
10	Lobule III of vermis	39	3.84	1	0.00016	4	−39	−9

Abbreviations: NIID, neuronal intranuclear inclusion disease; HC, healthy controls; GMV, grey matter volume; FWE, family-wise error.

At an exploratory, uncorrected threshold (*P* < 0.001, cluster extent ≥30 voxels), the NIID group showed increased GMV in the cerebellar vermis Lobule III and right middle frontal gyrus compared to HCs. This paradoxical increase in GMV may reflect underlying processes such as gliosis, neuroinflammation or compensatory cellular changes. Considering that it did not survive FWE correction, this finding should be interpreted with caution.

### Cortical thickness analysis

**Figure 2:**
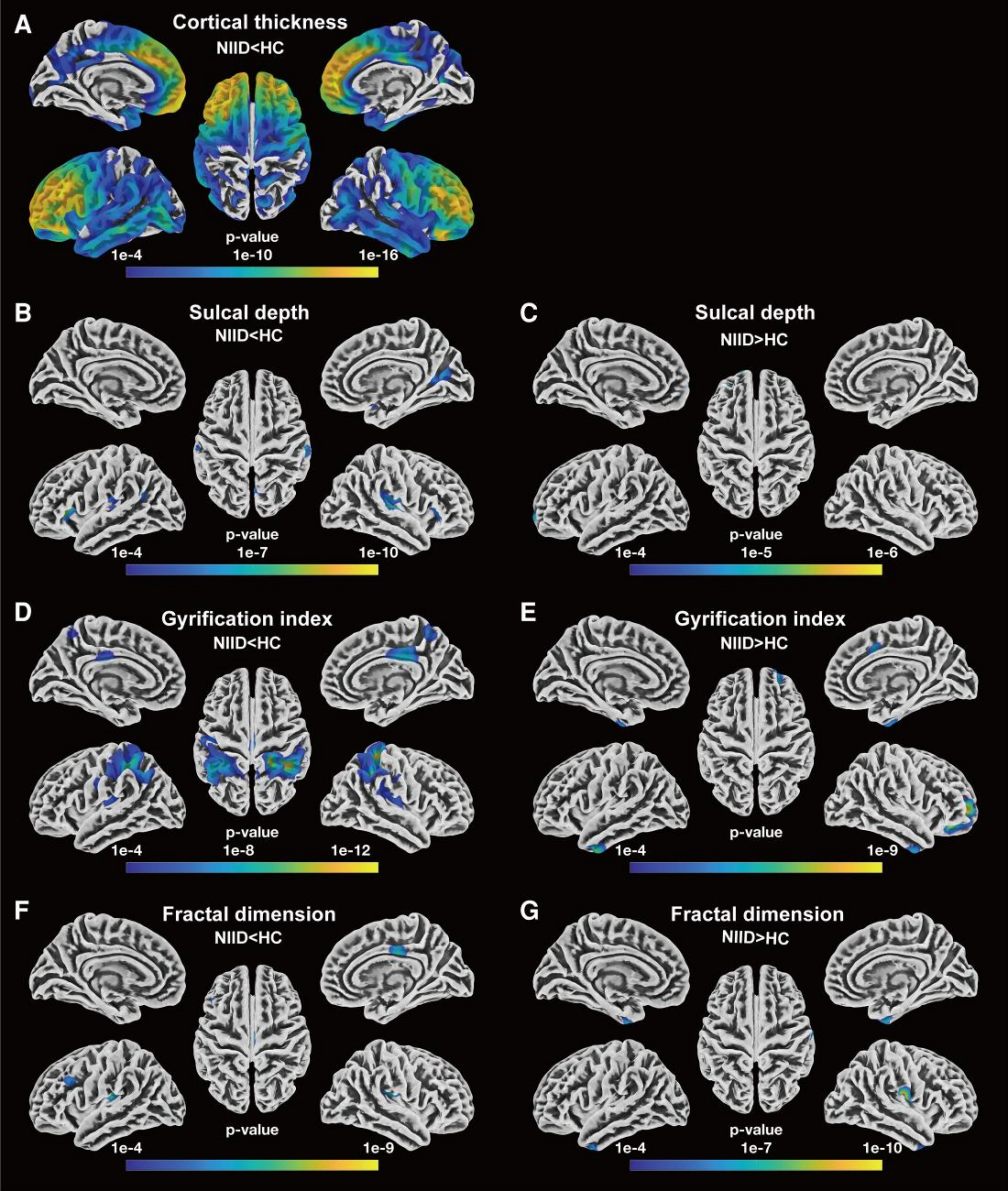
**Cortical architecture alterations in NIID.** SBM analysis of cortical architecture alterations in NIID patients versus healthy controls. (**A**) Cortical thickness (NIID < HC). (**B**) Sulcal depth (NIID < HC). (**C**) Sulcal depth (NIID > HC). (**D**) Gyrification index (NIID < HC). (**E**) Gyrification index (NIID > HC). (**F**) Fractal dimension (NIID < HC). (**G**) Fractal dimension (NIID > HC). Analyses were conducted using two-sample *t*-tests with FWE correction at *P* < 0.05, adjusting for sex and age (NIID *N* = 41; HC *N* = 21). NIID, neuronal intranuclear inclusion disease; HC, healthy controls.

**Figure 3:**
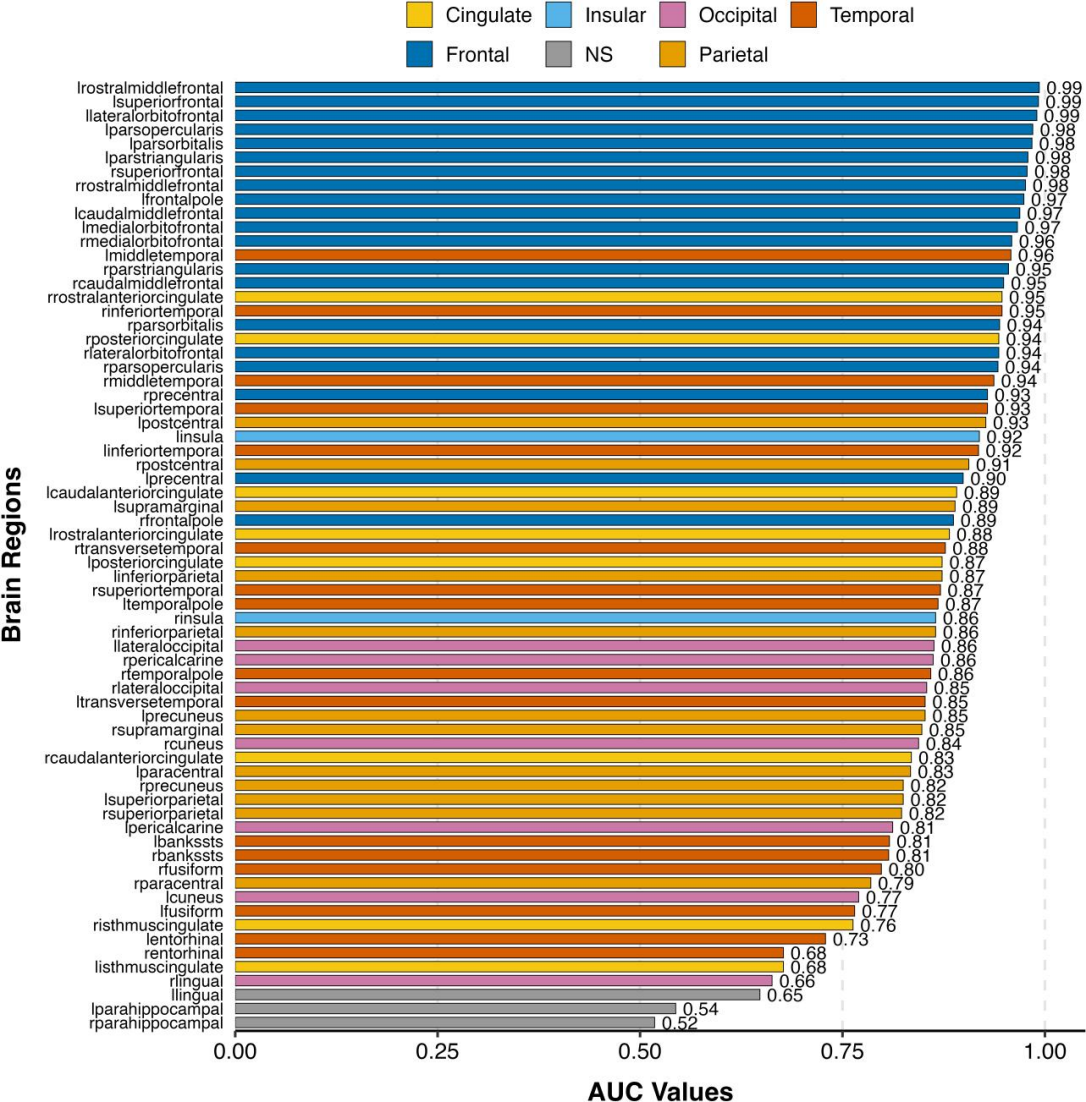
**Regional cortical thickness differences.** Region of interest analysis of cortical thickness differences between NIID patients and healthy controls using the Desikan–Killiany atlas. Areas under the curve (AUC) from receiver operating characteristic analyses indicate each region’s ability to discriminate between groups. Regions are ranked by descending AUC and color-coded by lobe: frontal, parietal, temporal, occipital, insular and cingulate. Frontal lobe regions exhibit the highest AUC values, indicating significant atrophy, while regions without significant classification are labelled as NS (grey) (NIID *N* = 41; HC *N* = 21). AUC, area under the curve; NS, non-significant; l, left; r, right.

Further details of the SBM results are presented in Fig. 2 and Supplementary Table 2, with all findings obtained under FWE correction at a statistical threshold of *P* < 0.05. SBM analysis revealed significant cortical thinning in NIID patients compared to HCs. The pattern of cortical thinning closely mirrored the GMV reductions observed in the VBM analysis, with pronounced effects in frontal, parietal and temporal regions bilaterally. Specifically, the superior frontal, precentral and rostral middle frontal areas showed the most extensive thinning, consistent with VBM findings. This frontal thinning was further supported by the ROC analysis of cortical thickness, where the top-ranked regions for classification accuracy were predominantly located in the frontal cortex, as shown by the AUC values in Fig. 3. Additionally, cortical thinning was noted in the precuneus, lateral occipital and cingulate regions, highlighting the widespread structural alterations associated with NIID. These results collectively indicate that NIID is characterized by extensive grey matter atrophy and cortical thinning, particularly affecting frontal lobe structures, but also involving key parietal, temporal and insular regions. The bilateral and widespread nature of these structural changes suggests a diffuse impact of the disease on brain morphology.

### Sulcal depth analysis

Compared to HCs, NIID patients exhibited decreased SD in the bilateral frontal lobes (including lateral orbitofrontal gyrus, opercular part and pars triangularis), temporal-insular regions (including insula, superior temporal gyrus, transverse temporal gyrus and supramarginal gyrus), sensorimotor areas (precentral and postcentral gyri) and the left inferior parietal lobule. Additionally, decreased SD was observed in the medial surface surrounding the right parieto-occipital sulcus, including precuneus, pericalcarine cortex and cuneus.

Conversely, the NIID group demonstrated increased SD in the left rostral middle frontal gyrus, superior frontal gyrus and the frontal pole region.

### Gyrification index analysis

GI analysis revealed significantly reduced GI in NIID patients, predominantly affecting bilateral parietal lobes, insula and cingulate cortex. Specifically, the parietal lobe exhibited widespread involvement, with reduced GI in the bilateral postcentral gyrus, supramarginal gyrus, precuneus, superior parietal lobule, inferior parietal lobule and right paracentral lobule. The cingulate gyrus showed reduced GI mainly in the posterior cingulate and isthmus-cingulate regions. Additionally, portions of the bilateral precentral gyrus and the right superior temporal gyrus also exhibited decreased GI.

In contrast, the NIID group showed increased GI in certain regions of the temporal lobe (bilateral inferior temporal gyrus, fusiform gyrus and left middle temporal gyrus), frontal lobe (right rostral middle frontal gyrus, right lateral orbitofrontal cortex and right superior frontal gyrus) and the right caudal anterior cingulate cortex. These areas with increased GI were more diffusely distributed across temporal and frontal lobes, with more pronounced involvement in the right hemisphere.

### Fractal dimension analysis

FD analysis revealed significant differences between NIID patients and HCs, partially overlapping with GI findings. Decreased FD was observed in the bilateral insula, right posterior cingulate gyrus and left middle frontal gyrus, as well as bilateral supramarginal gyrus, postcentral gyrus and left superior parietal lobule. Conversely, increased FD was found in the bilateral fusiform gyrus, inferior temporal gyrus, temporal pole, right postcentral and precentral gyri, and left superior frontal gyrus.

### Association of GMV with WMH volume and CSF fraction in NIID patients

**Figure 4:**
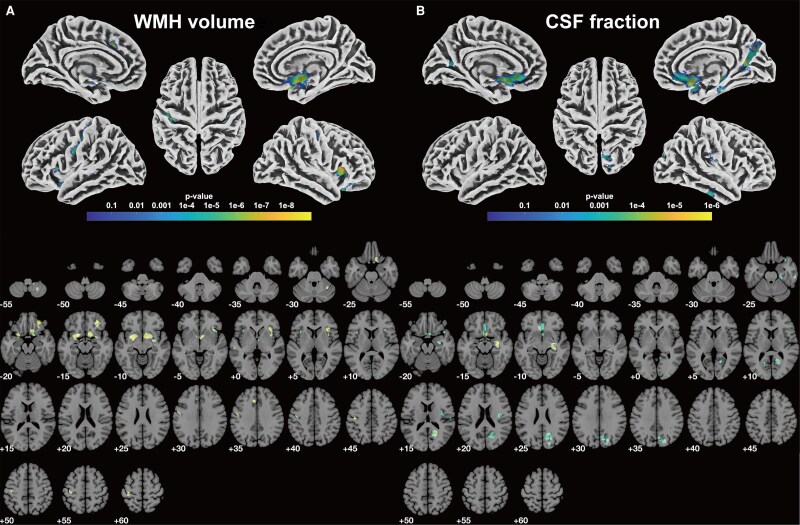
**Structural correlations with WMH volume and CSF fraction.** Brain atrophy associated with WMH volume and CSF fraction in NIID patients. (**A**) Regions of decreased GMV correlated with increased WMH volume (*P* < 0.05, family-wise error corrected). (**B**) Regions of decreased GMV correlated with increased CSF fraction (*P* < 0.001, minimum cluster size 30 voxels). Statistical associations were assessed using multiple regression analyses, adjusting for sex, age and total intracranial volume (NIID *N* = 41; HC *N* = 21). WMH, white matter hyperintensity; CSF, cerebrospinal fluid.

Fig. 4A and Table 3 further illustrate the spatial distribution of GMV associated with WMH volume. The results revealed a significant correlation between increased WMH volume and decreased GMV in multiple brain regions (FWE corrected, *P* < 0.05). The affected areas primarily included the frontal lobe, temporal lobe, insula, cingulate cortex, basal ganglia and cerebellum. Specifically, frontal involvement encompassed the orbitofrontal cortex (including bilateral olfactory cortices, posterior orbital gyri, right anterior orbital gyrus, right gyrus rectus and right medial orbital gyrus) and the triangular and opercular parts of the inferior frontal gyrus. Temporal impact was mainly seen in the left superior and middle temporal gyri, including the left temporal pole. Additionally, the analysis revealed bilateral involvement of the insula, precentral gyrus, postcentral gyrus and parts of the cingulate cortex (including middle cingulate and paracingulate gyri and anterior cingulate cortex). Subcortical structures such as the bilateral putamen, left amygdala and bilateral nucleus accumbens were also affected. Cerebellar involvement was primarily observed in the right hemisphere, including Lobule VIII, Crus I, Lobule VI and Lobule IX.

**Table 3:** Significant correlations of white matter hyperintensity volume with grey matter volume in NIID patients (FWE corrected, *P* < 0.05)

Overlap of atlas region		Cluster size	*r*	*P*-value	Peak MNI coordinates		
					*x*	*y*	*z*
42%	Unknown	785	0.8	9.10E−10	−24	0	−10
21%	Left lenticular nucleus-putamen						
13%	Left insula						
12%	Left olfactory cortex						
5%	Left amygdala						
3%	Left posterior orbital gyrus						
2%	Left nucleus accumbens						
2%	Left temporal pole: superior temporal gyrus						
25%	Unknown	1795	0.78	2.80E−09	32	40	−20
16%	Right anterior orbital gyrus						
12%	Right gyrus rectus						
11%	Right nucleus accumbens						
9%	Right medial orbital gyrus						
9%	Right olfactory cortex						
8%	Right lenticular nucleus-putamen						
7%	Right posterior orbital gyrus						
84%	Right insula	588	0.77	7.40E−09	34	22	6
6%	Unknown						
6%	Right inferior frontal gyrus-triangular part						
4%	Right inferior frontal gyrus-opercular part						
89%	Left middle cingulate and paracingulate gyri	122	0.76	1.80E−08	−8	22	36
11%	Left anterior cingulate cortex-supracallosal						
67%	Left postcentral gyrus	564	0.75	3.40E−08	−36	−30	62
33%	Left precentral gyrus						
60%	Left superior temporal gyrus	35	0.73	1.30E−07	−57	−26	2
40%	Left middle temporal gyrus						
54%	Right postcentral gyrus	39	0.71	2.80E−07	42	−12	48
46%	Right precentral gyrus						
73%	Left insula	130	0.7	4.90E−07	−34	16	6
22%	Left inferior frontal gyrus-opercular part						
5%	Left inferior frontal gyrus-triangular part						
89%	Right lobule VI of cerebellar hemisphere	56	0.7	5.70E−07	34	−52	−32
11%	Right crus I of cerebellar hemisphere						
75%	Right lobule IX of cerebellar hemisphere	59	0.7	6.10E−07	14	−42	−54
24%	Right lobule VIII of cerebellar hemisphere						
2%	Unknown						
100%	Right lobule VIII of cerebellar hemisphere	96	0.68	1.30E−06	24	−58	−54

We further conducted an exploratory analysis to examine the relationship between CSF fraction and GMV atrophy (uncorrected, *P* < 0.001, cluster extent threshold of 30 voxels) (Fig. 4B and Supplementary Table 3). Although no clusters survived FWE correction, preliminary negative correlations were observed predominantly in the right hippocampus and amygdala, bilateral anterior cingulate cortex, olfactory cortex and medial orbital superior frontal gyrus, as well as right posterior cortical regions (cuneus, precuneus and pericalcarine cortex), insula and the right temporal lobe. These trends align with the spatial distribution of atrophy but should be interpreted with caution given the uncorrected statistical nature of these findings.

### Exploratory correlations between brain morphometry and clinical features

**Figure 5:**
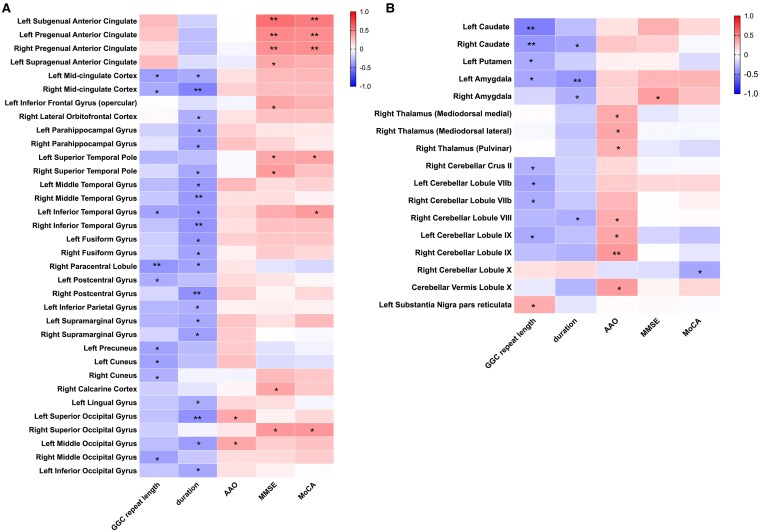
**Correlations between regional atrophy and clinical-genetic metrics.** Exploratory heatmaps of Spearman correlations between regional GMV and clinical-genetic profiles in patients with NIID. (**A**) Cortical regions categorized by anatomical systems (cingulate, frontal, temporal, parietal and occipital). (**B**) Subcortical nuclei and cerebellar regions. The heatmap illustrates Spearman’s rank correlation coefficients (ρ) between regional GMV and clinical variables (GGC repeat length, disease duration, AAO, MMSE and MoCA scores). Colour scale represents the correlation coefficient, with warmer and cooler tones indicating positive and negative correlations, respectively. Statistical associations were assessed using Spearman’s rank correlation analysis (*P < 0.05; **P < 0.01; NIID *N* = 41; HC *N* = 21). AAO, age at onset; MMSE, Mini-Mental State Examination; MoCA, Montreal Cognitive Assessment.

Spearman correlation analysis revealed distinct patterns of association between GMV and clinical-genetic profiles (Fig. 5). For genetic burden, GGC repeat length showed significant negative correlations with GMV in the bilateral middle cingulate cortex (MCC), bilateral caudate, left putamen, left amygdala, left postcentral gyrus, right paracentral lobule, left inferior temporal gyrus and multiple posterior regions including the bilateral cuneus, left precuneus and right middle occipital gyrus. Notably, cerebellar atrophy (Lobules VIIb, IX and Crus II) was also significantly linked to higher GGC repeats, whereas a paradoxical positive correlation was observed in the left substantia nigra pars reticulata.

Regarding disease progression, disease duration was negatively correlated with GMV in a broader network, including the bilateral MCC, bilateral amygdala, right caudate, right lateral orbitofrontal cortex, right postcentral gyrus, right paracentral lobule, left inferior parietal lobule, bilateral supramarginal gyrus, bilateral fusiform gyrus, right superior temporal pole, bilateral middle temporal gyrus, bilateral inferior temporal gyrus, bilateral parahippocampal gyrus, left lingual gyrus, left superior, middle and inferior occipital gyri and right cerebellar lobule VIII. AAO showed positive correlations with GMV in the right thalamus (mediodorsal and pulvinar nuclei), left superior and middle occipital gyrus and cerebellum regions (lobules VIII, IX and vermis X), suggesting a distinct vulnerability profile in early-onset phenotypes.

Cognitive performance (MMSE and MoCA) showed overlapping positive correlations with GMV in the anterior cingulate cortex, superior temporal pole and superior occipital gyrus. Specifically, MMSE scores correlated positively with GMV in right amygdala, left inferior frontal gyrus (opercular part) and right calcarine cortex, while MoCA scores correlated with GMV in left inferior temporal gyrus.

## Discussion

This study provides a comprehensive analysis of structural brain changes in patients with NIID using VBM and SBM. Our findings reveal significant GMV reductions, cortical thinning and complex morphological alterations, particularly in evolutionarily advanced regions such as the prefrontal cortex (PFC), cerebellum, insula and limbic system structures. The pronounced vulnerability of these regions in NIID suggests a potential link between genetic factors driving cortical expansion and susceptibility to neurodegenerative diseases. These findings provide new insights into the neuroanatomical basis of NIID and its potential implications for understanding the disease’s pathophysiology and clinical manifestations.

### Selective vulnerability of evolutionarily expanded networks

Our study revealed significant atrophy and cortical thinning in the PFC, with particularly prominent involvement of dorsolateral and ventromedial regions, alongside marked volume loss in cerebellar Crus I, Crus II and lobules VI, VIII, IX and X. These findings are particularly noteworthy considering the characteristic imaging features of PFC and the cerebellum in NIID and their unique development in the evolution of the human brain. In NIID, corticomedullary junction hyperintensities often originate in the frontal lobe, suggesting that this region may be an early site of pathological changes.^16^ Additionally, some NIID patients exhibit high signal intensities in the cerebellar paravermis and middle cerebellar peduncles on imaging, further supporting cerebellar involvement.^27^ Notably, the PFC and cerebellar Crus I/II are interconnected through cerebro-cerebellar circuits, and both have undergone dramatic expansion during human evolution, being disproportionately larger in humans compared to other primates.^2,28,29^ These areas underpin advanced cognitive functions, including decision-making, social behaviour and abstract reasoning.^2,29^

The observed pattern of regional vulnerability may be mechanistically linked to the developmental role of the human-specific *NOTCH2NL* gene family in cortical and cerebellar expansion. The *NOTCH2NL* gene family emerged approximately 3–4 million years ago through partial duplication of *NOTCH2*, coinciding with the dramatic cortical expansion in the hominin lineage.^10,11^ These genes enhance Notch signalling and regulate the proliferation and differentiation of neural progenitor cells, thereby promoting neocortical expansion, particularly in the prefrontal cortex.^3,5,10,11,30^ Furthermore, Notch signalling plays a crucial role in cerebellar development, regulating the proliferation and differentiation of granule cell precursors and maintaining the progenitor cell pool necessary for proper cerebellar layers and structure.^31^ Abnormal GGC repeat expansion in the 5′ untranslated region of *NOTCH2NLC* causes NIID, a progressive neurodegenerative disease characterized by widespread intranuclear inclusions.^12-15^ Proposed mechanisms include RNA toxicity from the expanded repeats sequestering RNA-binding proteins, production of toxic polyglycine (polyG) proteins via repeat-associated non-AUG (RAN) translation, disrupted protein function and downstream effects such as impaired nucleocytoplasmic transport, aberrant alternative splicing, nucleolar stress, mitochondrial dysfunction and neuroinflammation.^32-37^

We propose that brain regions most dependent on NOTCH2NL signalling during development, particularly those expanded in human evolution, may be selectively vulnerable when *NOTCH2NLC* function is disrupted by pathological repeat expansions. This aligns with the ‘last-in-first-out’ hypothesis, which suggests that recently evolved brain areas are more vulnerable to age-related and pathological changes.^38,39^ The PFC’s evolutionary expansion, characterized by increased dendritic arborization and synaptic connectivity, facilitates its complex integrative functions but may simultaneously increase metabolic demands and vulnerability to cellular stress. Similarly, the human cerebellum exhibits exceptional expansion, with the highest neuronal density among brain regions and pronounced human-specific methylation differences, which may underlie its vulnerability in NIID.^40-42^

### Complex cortical morphology changes

Our SBM analysis revealed complex alterations in cortical architecture beyond simple thinning. We observed decreased GI and FD in the bilateral parietal lobes, insula, posterior cingulate gyrus and parts of the frontal lobes, indicating reduced cortical complexity and folding in these regions.^43^ This simplification may result from neuronal loss and synaptic pruning, potentially affecting functions such as spatial orientation, attention, sensory integration and self-referential processing. Specifically, the parietal lobes play a crucial role in integrating sensory information and coordinating spatial awareness and their deterioration may underlie the cognitive deficits observed in NIID patients. The insula is integral to processing and integrating sensory and emotional information, while the posterior cingulate gyrus is a key component of the default mode network, involved in various cognitive functions such as memory retrieval and self-referential thinking.^44^ The concurrent involvement of these interconnected regions highlights the disruption of integrated brain networks in NIID, which may contribute to the widespread functional impairments.

Conversely, specific cortical areas, notably in the temporal lobe (inferior temporal gyrus and fusiform), right frontal lobe (rostral middle frontal gyrus, lateral orbitofrontal gyrus and superior frontal gyrus) and right caudal anterior cingulate, showed increased GI, with increased FD overlapping in fusiform, inferior temporal gyrus and superior frontal gyrus. These findings were complemented by explorative VBM results showing increased GMV in the right middle frontal gyrus and cerebellar vermis Lobule III. While increased complexity might suggest compensatory plasticity in other contexts, this interpretation requires caution in NIID. Neuropathologically, NIID is characterized not only by intranuclear inclusions but also by reactive astrocytosis, microglial activation and spongiotic changes,^45^ which may increase tissue water content or cellular density and thus inflate morphometric measures on T1-weighted MRI. Furthermore, the characteristic corticomedullary leucoencephalopathy may disrupt U-fibres, releasing axonal tension and causing the cortex to buckle mechanically, artificially elevating GI and FD. Additionally, *NOTCH2NLC*’s role in human cortical morphogenesis suggests that its pathogenic mutation may dysregulate developmental folding programmes, contributing to maladaptive structural complexity in frontal–temporal regions.

SD analysis further revealed complex bidirectional changes. Decreased SD was prominent in frontal, temporal-insular and sensorimotor regions, as well as medial parieto-occipital areas. This reduction likely reflects atrophy extending into the sulcal folds, leading to a shallower appearance or a loss of structural support from underlying white matter tracts (e.g. U-fibres) due to axonal loss or demyelination, which is particularly relevant given NIID’s characteristic corticomedullary junction pathology. Conversely, increased SD in anterior frontal regions might indicate localized compensatory folding or biomechanical tension from neighbouring tissue loss.

Taken together, these SBM findings highlight complex cortical reorganization in NIID, reflecting the interplay between primary neurodegenerative changes and potential secondary compensatory mechanisms. These processes might also be modulated by neurodevelopmental variations linked to *NOTCH2NLC*’s ancestral role in shaping cortical architecture.

### Insular cortex and limbic system alterations

The insula exhibited significant structural changes in NIID patients, including decreased GMV, cortical thinning and reductions in SD, GI and FD, indicating degeneration and simplification of this critical brain hub. The insula plays a crucial role in various functions, including interoception, emotional processing, pain perception, autonomic control and sensory integration.^46-48^ It maintains extensive connectivity with brain regions such as the anterior cingulate cortex, orbitofrontal cortex and amygdala, facilitating cognitive, emotional and executive functions.^49^

Alongside insular changes, there is notable involvement of the limbic system, with overlapping impacts of increased WMH volume and CSF fraction on grey matter atrophy, such as the anterior cingulate cortex, olfactory cortex, orbitofrontal cortex, insula, amygdala and temporal pole. This overlap reflects the vulnerability of these areas in the pathology of NIID and the potential importance of the limbic system in NIID. These regions are integral to emotion regulation, memory formation and autonomic functions.^50^ Their degeneration may underlie the neuropsychiatric symptoms associated with NIID, such as mood disturbances and memory impairment.

Notably, regions rich in Von Economo neurons, such as the insula and anterior cingulate cortex, are particularly affected. These neurons are thought to have evolved primarily in primates, are particularly vulnerable in neuropsychiatric disorders with impaired social and emotional functions and may play a key role in autonomic regulation.^51^

### Clinical and genetic associations

Our exploratory correlation analyses provide preliminary evidence linking structural damage to genetic burden and clinical features. First, the negative correlation between GGC repeat length and GMV in the MCC, striatum, sensorimotor cortex and cerebellum aligns with the high prevalence of movement disorders in NIID. This dose-dependent atrophy suggests that longer repeats may drive more aggressive neurodegeneration in these metabolically demanding nuclei, potentially through enhanced accumulation of RNA foci or RAN translation products. This pattern echoes findings in other repeat expansion disorders like striatal atrophy in Huntington’s disease and cerebellar degeneration in spinocerebellar ataxia.^52,53^ These localized structural vulnerabilities also provide an anatomical substrate for the large-scale functional network collapse previously reported in NIID, including disrupted connectivity across the default mode, sensorimotor, visual and salience networks.^54^ The association between disease duration and widespread structural alterations underscores the transition from focal insults to a more diffuse, multisystem neurodegeneration over time. Intriguingly, an earlier AAO was linked to greater atrophy in the thalamus (mediodorsal and pulvinar nuclei), occipital lobe and cerebellum. The mediodorsal thalamus is a key node in circuits governing consciousness and cognitive regulation, whereas the pulvinar and occipital cortices are central to visual information processing. The preferential vulnerability of these distinct regions may provide a structural basis for the episodic encephalopathy and visual symptoms, which are increasingly observed in case reports of young NIID patients.^55,56^ Furthermore, the positive correlation between cognitive scores (MMSE/MoCA) and the GMV in anterior cingulate cortex, superior occipital gyrus and superior temporal pole defines the structural epicentre of NIID-related dementia. Unlike Alzheimer’s disease, which primarily targets the hippocampus, NIID-related cognitive decline is more closely tied to the disintegration of the salience network, visuospatial and semantic hubs, explaining the executive, attentional and behavioural deficits. This interpretation is compatible with recent voxel-wise analysis of abnormal tau deposition in the occipital lobe and temporal pole in NIID, which may contribute to region-specific cognitive vulnerability.^57^ Notably, these analyses are exploratory and require replication in larger, longitudinal cohorts with comprehensive motor and autonomic scores to better resolve symptom-subtype specificity.

### Unique neuroimaging profile of NIID

The observed morphometric profile in NIID shares some similarities with other neurodegenerative diseases yet remains distinct in its distribution and pattern. Alzheimer’s disease typically manifests with medial temporal and parietal atrophy, especially within the default mode network. Parkinson’s disease primarily involves nigrostriatal pathways. While leucodystrophies involve extensive white matter changes, they rarely exhibit the specific, concomitant atrophy of the cerebellum and insula seen in our cohort. The unique constellation of extensive corticomedullary WMH combined with selective atrophy of the PFC, cerebellum and Von Economo neuron-rich insula distinguishes NIID from other neurodegenerative disorders. This specificity supports the notion that *NOTCH2NLC* mutations do not merely cause generalized atrophy but target specific neural networks defined by their genetic and evolutionary identity. This distinct profile aligns with the *post hoc* hypothesis of evolutionary vulnerability that the genetic mechanisms driving human cortical expansion may also confer selective vulnerability to neurodegeneration when dysregulated.

### Limitations

This study has limitations. The relatively small sample size may limit the statistical power of our analyses. Given the disease’s heterogeneity, future studies should involve larger sample sizes and stratified analyses to explore the relationship between structural changes and specific clinical symptoms in NIID. Additionally, as a cross-sectional study, it is limited in observing the dynamic progression of the disease. Longitudinal studies are needed to elucidate disease progression and structural changes over time. Finally, this study primarily focused on structural MRI and did not analyse diffusion MRI metrics. Although WMH volume captures macroscopic leucoencephalopathy, it lacks sensitivity to microstructural disintegration, particularly in normal-appearing white matter where early degeneration often precedes visible lesions. Recent studies have demonstrated that diffusion MRI metrics provide a more sensitive biomarker for early white matter integrity loss.^58-60^ Future studies should incorporate multimodal imaging approaches, including DTI, to comprehensively elucidate the interplay between white matter microstructure and cortical morphometry in NIID.

## Conclusions

In summary, *NOTCH2NLC*-related NIID exhibits a distinct pattern of grey matter loss and cortical complexity alterations, predominantly affecting evolutionarily expanded prefrontal–cerebellar and limbic–insular circuits. These findings suggest that human-specific genetic innovations, such as those involving *NOTCH2NLC*, not only shaped cortical architecture but may also confer selective vulnerability to degeneration. This *post hoc*, hypothesis-generating interpretation provides a framework for integrating molecular genetics, neuroanatomy and human evolution in the study of NIID. Future research should prioritize longitudinal designs and larger cohorts to validate these patterns and their clinical implications.

## Supplementary Material

fcag206_Supplementary_Data

## Data Availability

The data for this study is not publicly available due to privacy and ethical constraints. The corresponding author can provide the data for this study upon reasonable request. All neuroimaging preprocessing and analyses were performed using standard software (SPM12 and the CAT12 toolbox within the MATLAB environment). No custom scripts or original codes were generated for data analysis in this study. Instead, analyses were conducted using the standard Graphical User Interface (GUI). To ensure full reproducibility, all specific parameters, smoothing kernels and statistical thresholds have been detailed within the Methods section.

## References

[1] Levy R. The prefrontal cortex: From monkey to man. Brain. 2024;147(3):794–815. 37972282 10.1093/brain/awad389 PMC10907097

[2] Preuss TM, Wise SP. Evolution of prefrontal cortex. Neuropsychopharmacology. 2022;47(1):3–19. 34363014 10.1038/s41386-021-01076-5 PMC8617185

[3] Florio M, Heide M, Pinson A, et al Evolution and cell-type specificity of human-specific genes preferentially expressed in progenitors of fetal neocortex. Elife. 2018;7:e32332. 10.7554/eLife.32332 PMC5898914 29561261

[4] Namba T, Huttner WB. What makes us human: Insights from the evolution and development of the human neocortex. Annu Rev Cell Dev Biol. 2024;40(1):427–452. 39356810 10.1146/annurev-cellbio-112122-032521

[5] Lodewijk GA, Fernandes DP, Vretzakis I, Savage JE, Jacobs FMJ. Evolution of human brain size-associated NOTCH2NL genes proceeds toward reduced protein levels. Mol Biol Evol. 2020;37(9):2531–2548. 32330268 10.1093/molbev/msaa104 PMC7475042

[6] Heide M, Huttner WB. Human-specific genes, cortical progenitor cells, and microcephaly. Cells. 2021;10(5):1209. 10.3390/cells10051209 PMC8156310 34063381

[7] Heide M, Haffner C, Murayama A, et al Human-specific ARHGAP11B increases size and folding of primate neocortex in the fetal marmoset. Science. 2020;369(6503):546–550. 32554627 10.1126/science.abb2401

[8] Florio M, Albert M, Taverna E, et al Human-specific gene ARHGAP11B promotes basal progenitor amplification and neocortex expansion. Science. 2015;347(6229):1465–1470. 25721503 10.1126/science.aaa1975

[9] Charrier C, Joshi K, Coutinho-Budd J, et al Inhibition of SRGAP2 function by its human-specific paralogs induces neoteny during spine maturation. Cell. 2012;149(4):923–935. 22559944 10.1016/j.cell.2012.03.034 PMC3357949

[10] Fiddes IT, Lodewijk GA, Mooring M, et al Human-specific NOTCH2NL genes affect notch signaling and cortical neurogenesis. Cell. 2018;173(6):1356–1369.e22. 29856954 10.1016/j.cell.2018.03.051 PMC5986104

[11] Suzuki IK, Gacquer D, Van Heurck R, et al Human-specific NOTCH2NL genes expand cortical neurogenesis through Delta/notch regulation. Cell. 2018;173(6):1370–1384.e16. 29856955 10.1016/j.cell.2018.03.067 PMC6092419

[12] Sone J, Mitsuhashi S, Fujita A, et al Long-read sequencing identifies GGC repeat expansions in NOTCH2NLC associated with neuronal intranuclear inclusion disease. Nat Genet. 2019;51(8):1215–1221. 31332381 10.1038/s41588-019-0459-y

[13] Ishiura H, Shibata S, Yoshimura J, et al Noncoding CGG repeat expansions in neuronal intranuclear inclusion disease, oculopharyngodistal myopathy and an overlapping disease. Nat Genet. 2019;51(8):1222–1232. 31332380 10.1038/s41588-019-0458-z

[14] Tian Y, Wang JL, Huang W, et al Expansion of human-specific GGC repeat in neuronal intranuclear inclusion disease-related disorders. Am J Hum Genet. 2019;105(1):166–176. 31178126 10.1016/j.ajhg.2019.05.013 PMC6612530

[15] Deng J, Gu M, Miao Y, et al Long-read sequencing identified repeat expansions in the 5'UTR of the NOTCH2NLC gene from Chinese patients with neuronal intranuclear inclusion disease. J Med Genet. 2019;56(11):758–764. 31413119 10.1136/jmedgenet-2019-106268

[16] Sone J, Mori K, Inagaki T, et al Clinicopathological features of adult-onset neuronal intranuclear inclusion disease. Brain. 2016;139(Pt 12):3170–3186. 27797808 10.1093/brain/aww249 PMC5382941

[17] Takahashi-Fujigasaki J. Neuronal intranuclear hyaline inclusion disease. Neuropathology. 2003;23(4):351–359. 14719553 10.1046/j.1440-1789.2003.00524.x

[18] Tai H, Wang A, Zhang Y, et al Clinical features and classification of neuronal intranuclear inclusion disease. Neurol Genet. 2023;9(2):e200057. 37090934 10.1212/NXG.0000000000200057 PMC10117695

[19] Tian Y, Zhou L, Gao J, et al Clinical features of NOTCH2NLC-related neuronal intranuclear inclusion disease. J Neurol Neurosurg Psychiatry. 2022;93(12):1289–1298. 36150844 10.1136/jnnp-2022-329772 PMC9685690

[20] Zeng T, Chen Y, Huang H, et al Neuronal intranuclear inclusion disease with NOTCH2NLC GGC repeat expansion: A systematic review and challenges of phenotypic characterization. Aging Dis. 2024;16(1):578–597. 38377026 10.14336/AD.2024.0131-1 PMC11745434

[21] Sone J, Kitagawa N, Sugawara E, et al Neuronal intranuclear inclusion disease cases with leukoencephalopathy diagnosed via skin biopsy. J Neurol Neurosurg Psychiatry. 2014;85(3):354–356. 24039026 10.1136/jnnp-2013-306084

[22] Mao C, Zhou L, Li J, et al Clinical-neuroimaging-pathological relationship analysis of adult onset neuronal intranuclear inclusion disease (NIID). BMC Neurol. 2022;22(1):486. 36522621 10.1186/s12883-022-03025-1 PMC9753287

[23] Ashburner J, Friston KJ. Voxel-based morphometry–the methods. Neuroimage 2000;11(6 Pt 1):805–821. 10860804 10.1006/nimg.2000.0582

[24] Dahnke R, Yotter RA, Gaser C. Cortical thickness and central surface estimation. Neuroimage. 2013;65:336–348. 23041529 10.1016/j.neuroimage.2012.09.050

[25] Ghirelli A, Tafuri B, Urso D, et al Cortical signature of depressive symptoms in frontotemporal dementia: A surface-based analysis. Ann Clin Transl Neurol. 2023;10(10):1704–1713. 37522381 10.1002/acn3.51860 PMC10578898

[26] Desikan RS, Ségonne F, Fischl B, et al An automated labeling system for subdividing the human cerebral cortex on MRI scans into gyral based regions of interest. Neuroimage. 2006;31(3):968–980. 16530430 10.1016/j.neuroimage.2006.01.021

[27] Sugiyama A, Sato N, Kimura Y, et al MR imaging features of the cerebellum in adult-onset neuronal intranuclear inclusion disease: 8 cases. AJNR Am J Neuroradiol. 2017;38(11):2100–2104. 28818825 10.3174/ajnr.A5336 PMC7963582

[28] Balsters JH, Cussans E, Diedrichsen J, et al Evolution of the cerebellar cortex: The selective expansion of prefrontal-projecting cerebellar lobules. Neuroimage. 2010;49(3):2045–2052. 19857577 10.1016/j.neuroimage.2009.10.045 PMC6436533

[29] Donahue CJ, Glasser MF, Preuss TM, Rilling JK, Van Essen DC. Quantitative assessment of prefrontal cortex in humans relative to nonhuman primates. Proc Natl Acad Sci U S A. 2018;115(22):E5183–eE5192. 29739891 10.1073/pnas.1721653115 PMC5984508

[30] Vanderhaeghen P, Polleux F. Developmental mechanisms underlying the evolution of human cortical circuits. Nat Rev Neurosci. 2023;24(4):213–232. 36792753 10.1038/s41583-023-00675-z PMC10064077

[31] Adachi T, Miyashita S, Yamashita M, et al Notch signaling between cerebellar granule cell progenitors. eNeuro. 2021;8(3):ENEURO.0468-20. 10.1523/ENEURO.0468-20.2021 PMC8121261 33762301

[32] Deng J, Zhou B, Yu J, et al Genetic origin of sporadic cases and RNA toxicity in neuronal intranuclear inclusion disease. J Med Genet. 2022;59(5):462–469. 33766934 10.1136/jmedgenet-2020-107649

[33] Boivin M, Deng J, Pfister V, et al Translation of GGC repeat expansions into a toxic polyglycine protein in NIID defines a novel class of human genetic disorders: The polyG diseases. Neuron. 2021;109(11):1825–1835.e5. 33887199 10.1016/j.neuron.2021.03.038 PMC8186563

[34] Zhong S, Lian Y, Luo W, et al Upstream open reading frame with NOTCH2NLC GGC expansion generates polyglycine aggregates and disrupts nucleocytoplasmic transport: Implications for polyglycine diseases. Acta Neuropathol. 2021;142(6):1003–1023. 34694469 10.1007/s00401-021-02375-3

[35] Liu Q, Zhang K, Kang Y, et al Expression of expanded GGC repeats within NOTCH2NLC causes behavioral deficits and neurodegeneration in a mouse model of neuronal intranuclear inclusion disease. Sci Adv. 2022;8(47):eadd6391. 36417528 10.1126/sciadv.add6391 PMC9683706

[36] Fan Y, Li MJ, Yang J, et al GGC repeat expansion in NOTCH2NLC induces dysfunction in ribosome biogenesis and translation. Brain. 2023;146(8):3373–3391. 36825461 10.1093/brain/awad058

[37] Yu J, Liufu T, Zheng Y, et al CGG repeat expansion in NOTCH2NLC causes mitochondrial dysfunction and progressive neurodegeneration in Drosophila model. Proc Natl Acad Sci U S A. 2022;119(41):e2208649119. 36191230 10.1073/pnas.2208649119 PMC9565157

[38] Vickery S, Patil KR, Dahnke R, et al The uniqueness of human vulnerability to brain aging in great ape evolution. Sci Adv. 2024;10(35):eado2733. 39196942 10.1126/sciadv.ado2733 PMC11352902

[39] Fjell AM, McEvoy L, Holland D, Dale AM, Walhovd KB. What is normal in normal aging? Effects of aging, amyloid and Alzheimer's disease on the cerebral cortex and the hippocampus. Prog Neurobiol. 2014;117:20–40. 24548606 10.1016/j.pneurobio.2014.02.004 PMC4343307

[40] Wagner MJ, Kim TH, Savall J, Schnitzer MJ, Luo L. Cerebellar granule cells encode the expectation of reward. Nature 2017;544(7648):96–100. 28321129 10.1038/nature21726 PMC5532014

[41] De Zeeuw CI, Lisberger SG, Raymond JL. Diversity and dynamism in the cerebellum. Nat Neurosci. 2021;24(2):160–167. 33288911 10.1038/s41593-020-00754-9

[42] Guevara EE, Hopkins WD, Hof PR, Ely JJ, Bradley BJ, Sherwood CC. Comparative analysis reveals distinctive epigenetic features of the human cerebellum. PLoS Genet. 2021;17(5):e1009506. 33956822 10.1371/journal.pgen.1009506 PMC8101944

[43] Ziukelis ET, Mak E, Dounavi ME, Su L, J TOB. Fractal dimension of the brain in neurodegenerative disease and dementia: A systematic review. Ageing Res Rev. 2022;79:101651. 35643264 10.1016/j.arr.2022.101651

[44] Lin P, Yang Y, Jovicich J, et al Static and dynamic posterior cingulate cortex nodal topology of default mode network predicts attention task performance. Brain Imaging Behav. 2016;10(1):212–225. 25904156 10.1007/s11682-015-9384-6

[45] Yoshii D, Ayaki T, Wada T, et al An autopsy case of adult-onset neuronal intranuclear inclusion disease with perivascular preservation in cerebral white matter. Neuropathology. 2022;42(1):66–73. 34954850 10.1111/neup.12778

[46] Gogolla N. The insular cortex. Curr Biol. 2017;27(12):R580–R586. 28633023 10.1016/j.cub.2017.05.010

[47] Zhang R, Deng H, Xiao X. The insular Cortex: An interface between sensation, emotion and cognition. Neurosci Bull. 2024;40(11):1763–1773. 38722464 10.1007/s12264-024-01211-4 PMC11607240

[48] Benarroch EE. Insular cortex: Functional complexity and clinical correlations. Neurology. 2019;93(21):932–938. 31645470 10.1212/WNL.0000000000008525

[49] Wang R, Mo F, Shen Y, Song Y, Cai H, Zhu J. Functional connectivity gradients of the insula to different cerebral systems. Hum Brain Mapp. 2023;44(2):790–800. 36206289 10.1002/hbm.26099 PMC9842882

[50] Catani M, Dell'acqua F, Thiebaut de Schotten M. A revised limbic system model for memory, emotion and behaviour. Neurosci Biobehav Rev. 2013;37(8):1724–1737. 23850593 10.1016/j.neubiorev.2013.07.001

[51] Butti C, Santos M, Uppal N, Hof PR. Von Economo neurons: Clinical and evolutionary perspectives. Cortex. 2013;49(1):312–326. 22130090 10.1016/j.cortex.2011.10.004

[52] Wang X, Li Y, Li B, Shang H, Yang J. Gray matter alterations in Huntington's disease: A meta-analysis of VBM neuroimaging studies. J Neurosci Res. 2024;102(7):e25366. 38953592 10.1002/jnr.25366

[53] Ye ZX, Chen XY, Li MC, et al Associations between CAG repeat size, brain and spinal cord volume loss, and motor symptoms in spinocerebellar ataxia type 3: A cohort study. Orphanet J Rare Dis. 2025;20(1):35. 39849568 10.1186/s13023-025-03531-8 PMC11761751

[54] Zhu R, Qu J, Wu Y, Xu G, Wang D. Diffusion and functional MRI reveal microstructural and network connectivity impairment in adult-onset neuronal intranuclear inclusion disease. Front Aging Neurosci. 2024;16:1478065. 39463819 10.3389/fnagi.2024.1478065 PMC11502314

[55] Xiao F, Tian X, Wang XF. Cerebral atrophy and leukoencephalopathy in a young man presenting with encephalitic episodes. JAMA Neurol. 2018;75(12):1563–1564. 30167633 10.1001/jamaneurol.2018.2333

[56] Su N, Mao HJ, Mao CH, et al Recurrent headache and visual symptoms in a young man: A rare neuronal intranuclear inclusion disease case report. BMC Neurol. 2022;22(1):401. 36324076 10.1186/s12883-022-02936-3 PMC9628060

[57] Zhang S, Jiao B, Zeng Y, et al Plasma p-tau species are elevated in presymptomatic and symptomatic neuronal intranuclear inclusion disease. EBioMedicine 2026;124:106127. 41539185 10.1016/j.ebiom.2026.106127 PMC12830138

[58] Uchida Y, Onda K, Nishimaki K, et al Longitudinal changes in brain diffusion characteristics associated with cognition and vascular risk factors: The ARIC-NCS study. Neurology. 2025;105(4):e213867. 40705997 10.1212/WNL.0000000000213867 PMC12296637

[59] Uchida Y, Hou Z, Gomez-Isaza L, et al Quantification of perforant path fibers for early detection of Alzheimer's disease. Alzheimers Dement 2025;21(4):e70142. 40189812 10.1002/alz.70142 PMC11972979

[60] Uchida Y, Onda K, Hou Z, Troncoso JC, Mori S, Oishi K. Microstructural neurodegeneration of the entorhinal-hippocampus pathway along the Alzheimer's disease Continuum. J Alzheimers Dis. 2023;95(3):1107–1117. 37638442 10.3233/JAD-230452 PMC10578220

