# Supplementary material for: Evolutionary implications of NOTCH2NLC mutations: brain structural changes in neuronal intranuclear inclusion disease revealed by comprehensive morphometry
Source: Brain Commun. 2026 Jun 3;8(4):fcag206. doi: 10.1093/braincomms/fcag206 (PMC13326951; doi:10.1093/braincomms/fcag206)
Supplement: fcag206_Supplementary_Data [file fcag206_supplementary_data.docx]

**Supplementary material**

**Supplementary Table 1: Detailed atlas region composition of each VBM cluster**

| **Cluster** | **Overlap (%)** | **Atlas region** | |
| --- | --- | --- | --- |
| 1 | 6% | Unknown |  |
|  | 3% | Left Postcentral gyrus |  |
|  | 3% | Left Middle cingulate & paracingulate gyri |  |
|  | 3% | Left Crus I of cerebellar hemisphere |  |
|  | 3% | Left Superior frontal gyrus-medial |  |
|  | 3% | Left Crus II of cerebellar hemisphere |  |
|  | 2% | Right Lobule VI of cerebellar hemisphere |  |
|  | 2% | Right Crus I of cerebellar hemisphere |  |
|  | 2% | Right Lobule VIII of cerebellar hemisphere |  |
|  | 2% | Right Crus II of cerebellar hemisphere |  |
|  | 2% | Left Middle temporal gyrus |  |
|  | 2% | Right Middle cingulate & paracingulate gyri |  |
|  | 2% | Left Lobule VI of cerebellar hemisphere |  |
|  | 2% | Right Insula |  |
|  | 2% | Left Insula |  |
|  | 2% | Left Superior temporal gyrus |  |
|  | 2% | Right Postcentral gyrus |  |
|  | 2% | Right Calcarine fissure and surrounding cortex |  |
|  | 1% | Left Precuneus |  |
|  | 1% | Left Supplementary motor area |  |
|  | 1% | Left Hippocampus |  |
|  | 1% | Left Lobule VIII of cerebellar hemisphere |  |
|  | 1% | Right Superior temporal gyrus |  |
|  | 1% | Left Lenticular nucleus-Putamen |  |
|  | 1% | Left Inferior frontal gyrus-triangular part |  |
|  | 1% | Right Hippocampus |  |
|  | 1% | Right Superior frontal gyrus-medial |  |
|  | 1% | Left Calcarine fissure and surrounding cortex |  |
|  | 1% | Left Anterior cingulate cortex-pregenual |  |
|  | 1% | Left Rolandic operculum |  |
|  | 1% | Right Inferior frontal gyrus-triangular part |  |
|  | 1% | Right Precuneus |  |
|  | 1% | Right Middle temporal gyrus |  |
|  | 1% | Right Superior frontal gyrus-medial orbital |  |
|  | 1% | Right Rolandic operculum |  |
| 2 | 54% | Left Middle frontal gyrus |  |
|  | 46% | Left Superior frontal gyrus-dorsolateral |  |
| 3 | 56% | Left Inferior temporal gyrus |  |
|  | 27% | Left Temporal pole: middle temporal gyrus |  |
|  | 11% | Unknown |  |
|  | 6% | Left Middle temporal gyrus |  |
| 4 | 89% | Left Superior frontal gyrus-dorsolateral |  |
|  | 4% | Left Superior frontal gyrus-medial orbital |  |
|  | 4% | Unknown |  |
|  | 3% | Left Anterior orbital gyrus |  |
| 5 | 91% | Right Angular gyrus |  |
|  | 5% | Right Inferior parietal gyrus-excluding supramarginal and angular gyri |  |
|  | 3% | Right Middle temporal gyrus |  |
| 6 | 88% | Left Postcentral gyrus |  |
|  | 10% | Left Paracentral lobule |  |
|  | 2% | Left Precentral gyrus |  |
| 7 | 59% | Right Lobule IX of cerebellar hemisphere |  |
|  | 23% | Unknown |  |
|  | 18% | Right Lobule X of cerebellar hemisphere |  |
| 8 | 59% | Right Superior frontal gyrus-dorsolateral |  |
|  | 41% | Right Middle frontal gyrus |  |
| 9 | 100% | Right Middle frontal gyrus |  |
| 10 | 56% | Lobule III of vermis |  |
|  | 44% | Unknown |  |

**Supplementary Table 2: Surface-based morphometry results: Brain regions showing significant changes in cortical thickness, sulcal depth, gyrification index, and fractal dimension in NIID patients compared to healthy controls (all results *p* < 0.05, FWE corrected).**

| **Hemisphere** | **Overlap of atlas region** | | **Cluster size** | **Cohen’s d** | ***p*-value** |
| --- | --- | --- | --- | --- | --- |
| **Cortical thickness: NIID<HC** | | | | | |
| LH | 12% | superiorfrontal | 20110 | 3.4 | <0.00001 |
|  | 8% | precentral |  |  |  |
|  | 8% | rostralmiddlefrontal |  |  |  |
|  | 6% | superiortemporal |  |  |  |
|  | 6% | supramarginal |  |  |  |
|  | 6% | inferiorparietal |  |  |  |
|  | 5% | postcentral |  |  |  |
|  | 5% | insula |  |  |  |
|  | 4% | middletemporal |  |  |  |
|  | 4% | lateralorbitofrontal |  |  |  |
|  | 4% | precuneus |  |  |  |
|  | 4% | caudalmiddlefrontal |  |  |  |
|  | 4% | lateraloccipital |  |  |  |
|  | 3% | parsopercularis |  |  |  |
|  | 3% | inferiortemporal |  |  |  |
|  | 2% | medialorbitofrontal |  |  |  |
|  | 2% | parstriangularis |  |  |  |
|  | 2% | paracentral |  |  |  |
|  | 2% | posteriorcingulate |  |  |  |
|  | 1% | rostralanteriorcingulate |  |  |  |
|  | 1% | superiorparietal |  |  |  |
|  | 1% | caudalanteriorcingulate |  |  |  |
|  | 1% | bankssts |  |  |  |
| LH | 100% | superiorparietal | 299 | 1.5 | <0.00001 |
| RH | 12% | superiorfrontal | 19028 | 3.1 | <0.00001 |
|  | 10% | precentral |  |  |  |
|  | 8% | rostralmiddlefrontal |  |  |  |
|  | 7% | postcentral |  |  |  |
|  | 6% | superiortemporal |  |  |  |
|  | 5% | middletemporal |  |  |  |
|  | 4% | insula |  |  |  |
|  | 4% | inferiortemporal |  |  |  |
|  | 4% | supramarginal |  |  |  |
|  | 4% | inferiorparietal |  |  |  |
|  | 4% | caudalmiddlefrontal |  |  |  |
|  | 3% | lateralorbitofrontal |  |  |  |
|  | 3% | precuneus |  |  |  |
|  | 3% | posteriorcingulate |  |  |  |
|  | 3% | parsopercularis |  |  |  |
|  | 3% | parstriangularis |  |  |  |
|  | 2% | superiorparietal |  |  |  |
|  | 2% | medialorbitofrontal |  |  |  |
|  | 2% | paracentral |  |  |  |
|  | 2% | lateraloccipital |  |  |  |
|  | 1% | parsorbitalis |  |  |  |
|  | 1% | isthmuscingulate |  |  |  |
|  | 1% | rostralanteriorcingulate |  |  |  |
| RH | 32% | superiorparietal | 776 | 2.2 | <0.00001 |
|  | 22% | cuneus |  |  |  |
|  | 22% | precuneus |  |  |  |
|  | 18% | pericalcarine |  |  |  |
|  | 4% | lingual |  |  |  |
|  | 2% | lateraloccipital |  |  |  |
| RH | 100% | fusiform | 62 | 1.4 | <0.00001 |
| **Sulcal depth: NIID<HC** | | | | | |
| LH | 35% | insula | 1039 | 1.9 | <0.00001 |
|  | 14% | parsopercularis |  |  |  |
|  | 13% | parstriangularis |  |  |  |
|  | 13% | postcentral |  |  |  |
|  | 9% | supramarginal |  |  |  |
|  | 8% | precentral |  |  |  |
|  | 8% | lateralorbitofrontal |  |  |  |
| LH | 69% | superiortemporal | 177 | 1.5 | <0.00001 |
|  | 31% | transversetemporal |  |  |  |
| LH | 92% | inferiorparietal | 137 | 1.5 | <0.00001 |
|  | 8% | supramarginal |  |  |  |
| LH | 74% | precentral | 108 | 1.3 | 0.00001 |
|  | 26% | postcentral |  |  |  |
| RH | 21% | superiortemporal | 1722 | 1.9 | <0.00001 |
|  | 20% | supramarginal |  |  |  |
|  | 16% | insula |  |  |  |
|  | 11% | parstriangularis |  |  |  |
|  | 10% | postcentral |  |  |  |
|  | 8% | transversetemporal |  |  |  |
|  | 5% | parsopercularis |  |  |  |
|  | 4% | precentral |  |  |  |
|  | 3% | lateralorbitofrontal |  |  |  |
| RH | 32% | pericalcarine | 399 | 1.5 | <0.00001 |
|  | 29% | precuneus |  |  |  |
|  | 25% | cuneus |  |  |  |
|  | 9% | lingual |  |  |  |
|  | 5% | isthmuscingulate |  |  |  |
| RH | 100% | precentral | 50 | 1.2 | 0.00001 |
| **Sulcal depth: NIID>HC** | | | | | |
| LH | 51% | superiorfrontal | 37 | 1.2 | 0.00001 |
|  | 32% | rostralmiddlefrontal |  |  |  |
|  | 16% | frontalpole |  |  |  |
| **Gyrification index: NIID<HC** | | | | | |
| LH | 31% | postcentral | 2554 | 1.9 | <0.00001 |
|  | 31% | superiorparietal |  |  |  |
|  | 21% | supramarginal |  |  |  |
|  | 11% | inferiorparietal |  |  |  |
|  | 3% | precentral |  |  |  |
|  | 3% | precuneus |  |  |  |
| LH | 100% | insula | 228 | 1.4 | <0.00001 |
| LH | 92% | posteriorcingulate | 181 | 1.4 | <0.00001 |
|  | 4% | isthmuscingulate |  |  |  |
| RH | 43% | superiorparietal | 2176 | 2.4 | <0.00001 |
|  | 23% | supramarginal |  |  |  |
|  | 19% | inferiorparietal |  |  |  |
|  | 15% | postcentral |  |  |  |
| RH | 53% | supramarginal | 927 | 1.5 | <0.00001 |
|  | 33% | insula |  |  |  |
|  | 10% | postcentral |  |  |  |
|  | 3% | superiortemporal |  |  |  |
| RH | 73% | posteriorcingulate | 434 | 1.8 | <0.00001 |
|  | 24% | isthmuscingulate |  |  |  |
| RH | 100% | precuneus | 185 | 1.5 | <0.00001 |
| RH | 91% | paracentral | 74 | 1.3 | <0.00001 |
|  | 8% | postcentral |  |  |  |
|  | 1% | precentral |  |  |  |
| **Gyrification index: NIID>HC** | | | | | |
| LH | 77% | inferiortemporal | 145 | 1.6 | <0.00001 |
|  | 18% | fusiform |  |  |  |
|  | 6% | middletemporal |  |  |  |
| RH | 57% | rostralmiddlefrontal | 416 | 1.8 | <0.00001 |
|  | 43% | lateralorbitofrontal |  |  |  |
| RH | 88% | inferiortemporal | 155 | 1.4 | <0.00001 |
|  | 12% | fusiform |  |  |  |
| RH | 86% | superiorfrontal | 94 | 1.5 | <0.00001 |
|  | 14% | caudalanteriorcingulate |  |  |  |
| **Fractal dimension: NIID<HC** | | | | | |
| LH | 94% | insula | 176 | 1.6 | <0.00001 |
|  | 3% | postcentral |  |  |  |
|  | 2% | supramarginal |  |  |  |
| LH | 79% | rostralmiddlefrontal | 77 | 1.4 | <0.00001 |
|  | 21% | caudalmiddlefrontal |  |  |  |
| LH | 100% | superiorparietal | 41 | 1.3 | <0.00001 |
| RH | 48% | supramarginal | 249 | 1.8 | <0.00001 |
|  | 38% | insula |  |  |  |
|  | 14% | postcentral |  |  |  |
| RH | 100% | posteriorcingulate | 120 | 1.5 | <0.00001 |
| **Fractal dimension: NIID>HC** | | | | | |
| LH | 47% | fusiform | 103 | 1.6 | <0.00001 |
|  | 34% | inferiortemporal |  |  |  |
|  | 12% | entorhinal |  |  |  |
|  | 8% | temporalpole |  |  |  |
| LH | 100% | superiorfrontal | 31 | 1.2 | 0.00001 |
| RH | 64% | postcentral | 273 | 2.0 | <0.00001 |
|  | 29% | precentral |  |  |  |
|  | 4% | supramarginal |  |  |  |
|  | 3% | parsopercularis |  |  |  |
| RH | 50% | fusiform | 109 | 1.5 | <0.00001 |
|  | 39% | inferiortemporal |  |  |  |
|  | 9% | temporalpole |  |  |  |
|  | 2% | entorhinal |  |  |  |

Abbreviation: NIID = neuronal intranuclear inclusion disease; HC = healthy controls; LH = left hemisphere; RH = right hemisphere.

**Supplementary Table 3: Significant correlations of cerebrospinal fluid fraction with gray matter volume in NIID patients (uncorrected, *p* < 0.001).**

| **Overlap of atlas region** | | | **Cluster**  **size** | ***r*** | ***p*-value** | **Peak MNI coordinates** | | |
| --- | --- | --- | --- | --- | --- | --- | --- | --- |
|  |  |  |  |  |  | **X** | **Y** | **Z** |
| 75% | Right Hippocampus | 602 | | 0.67 | 2.20E-06 | 30 | -12 | -16 |
| 15% | Unknown |  | |  |  |  |  |  |
| 9% | Right Amygdala |  | |  |  |  |  |  |
| 1% | Right Lateral geniculate |  | |  |  |  |  |  |
| 26% | Left Anterior cingulate cortex-subgenual | 892 | | 0.64 | 6.30E-06 | 2 | 9 | -16 |
| 20% | Left Olfactory cortex |  | |  |  |  |  |  |
| 20% | Unknown |  | |  |  |  |  |  |
| 14% | Right Olfactory cortex |  | |  |  |  |  |  |
| 10% | Right Superior frontal gyrus-medial orbital |  | |  |  |  |  |  |
| 7% | Right Anterior cingulate cortex-subgenual |  | |  |  |  |  |  |
| 2% | Left Superior frontal gyrus-medial orbital |  | |  |  |  |  |  |
| 39% | Right Cuneus | 1943 | | 0.60 | 2.90E-05 | 20 | -78 | 34 |
| 31% | Right Precuneus |  | |  |  |  |  |  |
| 23% | Right Calcarine fissure and surrounding cortex |  | |  |  |  |  |  |
| 7% | Right Superior occipital gyrus |  | |  |  |  |  |  |
| 80% | Right Temporal pole: superior temporal gyrus | 120 | | 0.58 | 5.80E-05 | 42 | 15 | -20 |
| 12% | Right Posterior orbital gyrus |  | |  |  |  |  |  |
| 8% | Unknown |  | |  |  |  |  |  |
| 100% | Right Inferior temporal gyrus | 90 | | 0.56 | 0.00014 | 62 | -28 | -28 |
| 73% | Right Insula | 219 | | 0.54 | 0.00022 | 36 | -14 | 21 |
| 18% | Right Rolandic operculum |  | |  |  |  |  |  |
| 9% | Unknown |  | |  |  |  |  |  |
| 71% | Right Parahippocampal gyrus | 119 | | 0.54 | 0.00024 | 24 | -27 | -26 |
| 10% | Unknown |  | |  |  |  |  |  |
| 9% | Right Fusiform gyrus |  | |  |  |  |  |  |
| 8% | Right Lobule IV-V of cerebellar hemisphere |  | |  |  |  |  |  |
| 100% | Left Calcarine fissure and surrounding cortex | 63 | | 0.53 | 0.0003 | -18 | -64 | 12 |
| 33% | Unknown | 57 | | 0.53 | 0.00033 | 8 | -51 | 2 |
| 33% | Right Precuneus |  | |  |  |  |  |  |
| 19% | Lobule IV-V of vermis |  | |  |  |  |  |  |
| 11% | Right Lingual gyrus |  | |  |  |  |  |  |
| 4% | Right Calcarine fissure and surrounding cortex |  | |  |  |  |  |  |
| 82% | Right Superior temporal gyrus | 40 | | 0.52 | 0.0004 | 58 | -20 | 16 |
| 18% | Right Rolandic operculum |  | |  |  |  |  |  |
